# Nanoparticle-Doped Polydimethylsiloxane Fluid Enhances the Optical Performance of AlGaN-Based Deep-Ultraviolet Light-Emitting Diodes

**DOI:** 10.1186/s11671-019-3067-y

**Published:** 2019-07-15

**Authors:** Zhi Ting Ye, Yung-Min Pai, Chih-Hao Lin, Lung-Chien Chen, Hong Thai Nguyen, Hsiang-Chen Wang

**Affiliations:** 1 Department of Electro-Optical Engineering, National United University, 2, Lienda, Miaoli, 26063 Taiwan; 2 Department of Photonics, National Chiao Tung University, 1001 University Road, Hsinchu, 300 Taiwan; 3 Department of Electro-Optical Engineering, National Taipei University of Technology, No.1, Sec. 3, Chung-Hsiao E. Rd, Taipei, 10608 Taiwan; 4 Department of Mechanical Engineering and Advanced Institute of Manufacturing with High-tech Innovations, National Chung Cheng University, 168, University Rd., Min-Hsiung, Chia-Yi, 62102 Taiwan

**Keywords:** DUV-LEDs, PDMS, Nanoparticle, Light extraction efficiency, Flip chip

## Abstract

This paper proposes a new encapsulation structure for aluminum nitride-based deep UV light-emitting diodes (DUV-LEDs) and eutectic flip chips containing polydimethylsiloxane (PDMS) fluid doped with SiO_2_ nanoparticles (NPs) with a UV-transparent quartz hemispherical glass cover. Experimental results reveal that the proposed encapsulation structure has considerably higher light output power than the traditional one. The light extraction efficiency was increased by 66.49% when the forward current of the DUV-LED was 200 mA. Doping the PDMS fluid with SiO_2_ NPs resulted in higher light output power than that of undoped fluid. The maximum efficiency was achieved at a doping concentration of 0.2 wt%. The optical output power at 200 mA forward current of the encapsulation structure with NP doping of the fluid was 15% higher than that without NP doping. The optical output power of the proposed encapsulation structure was 81.49% higher than that of the traditional encapsulation structure. The enhanced light output power was due to light scattering caused by the SiO_2_ NPs and the increased average refractive index. The encapsulation temperature can be reduced by 4 °C at a driving current of 200 mA by using the proposed encapsulation structure.

## Background

Aluminum nitride-based deep UV-emitting diodes (DUV-LEDs) with a eutectic flip chip and a wavelength range of 200–300 nm have been used in curing engineering, communication security, sterilization engineering, chemical decomposition, water purification, air purification, forgery detection, and sensing [1–10]. DUV-LEDs are considered a near-future replacement for traditional UV light sources because they are free from mercury and highly reliable [11–14]. However, the output power of the flip chip DUV-LED remains low mainly because of quantum well defects, light absorption, and total internal reflection (TIR) at the sapphire–air interface [15–17]. The light extraction efficiency (LEE) of visible-light LEDs has been improved by reducing TIR loss using a silicon encapsulation layer [18–30]. In this paper, we propose a fluid encapsulation method by using polydimethylsiloxane (PDMS) with high refractive index (*n* = 1.43) and transmittance at a wavelength of 275 nm. The PDMS fluid has excellent properties, such as nontoxicity and resistance to oxidation, chemicals, and heat [31, 32]. The proposed encapsulation method enhances the light output efficiency of DUV-LEDs and reduces the adverse effects of LEDs on people and the environment. Mixing SiO_2_ NPs into the PDMS fluid can also improve the light efficiency.

## Methods and Materials

**Fig. 1:**
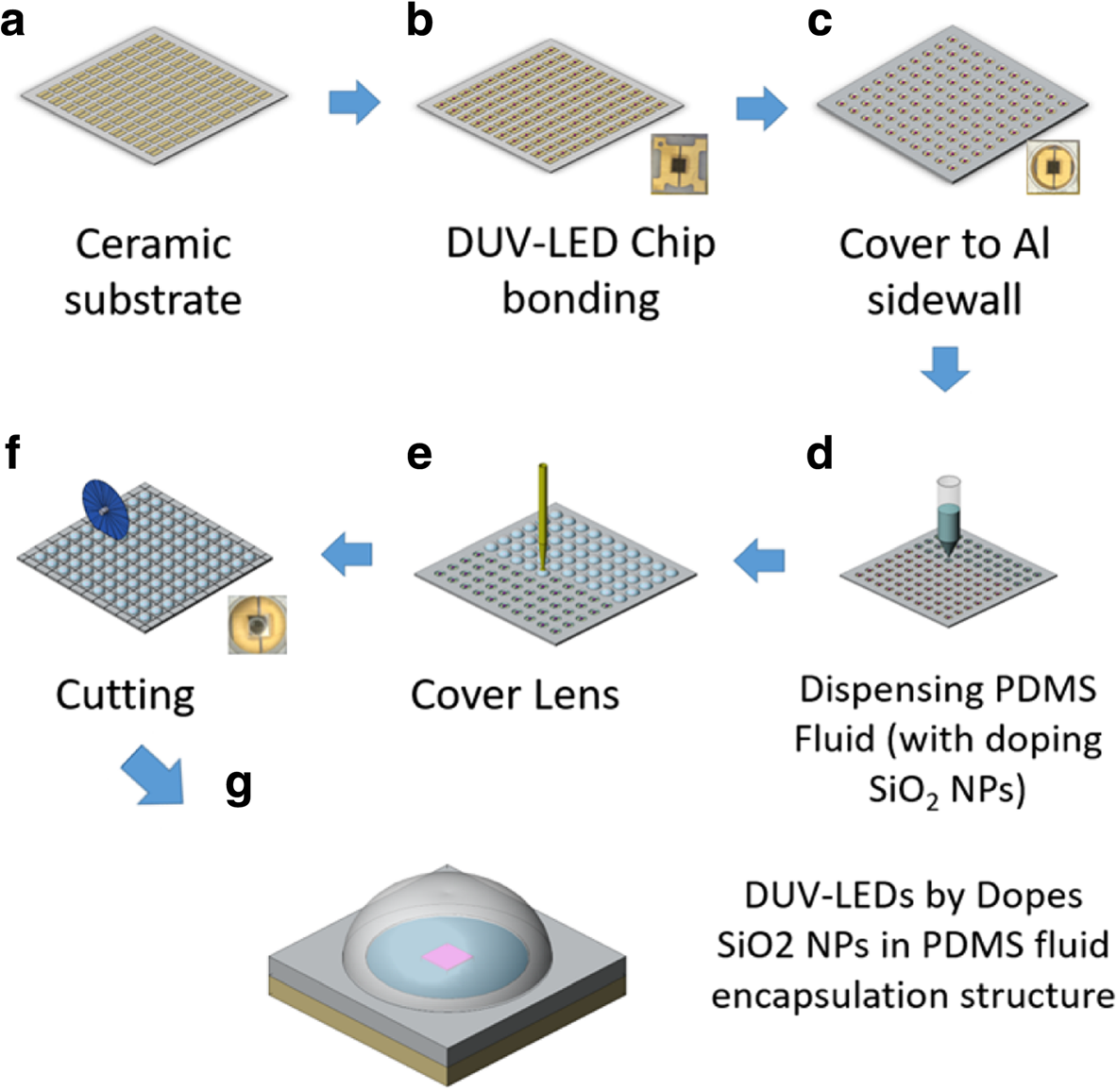
Fabrication of DUV-LED encapsulation structure: **a** ceramic substrate, **b** DUV-LED chip (peak wavelength, 275 nm) bonded to a ceramic substrate through pressure bonding, **c** aluminum plate bonded to the DUV-LED ceramic substrate, **d** doped binder dispensed into the cavity, **e** a quartz lens cover placed on the structure, **f** cut-out finished DUV-LEDs, and **g** complete DUV-LED with a SiO_2_-NP-doped PDMS fluid encapsulation structure

**Fig. 2:**
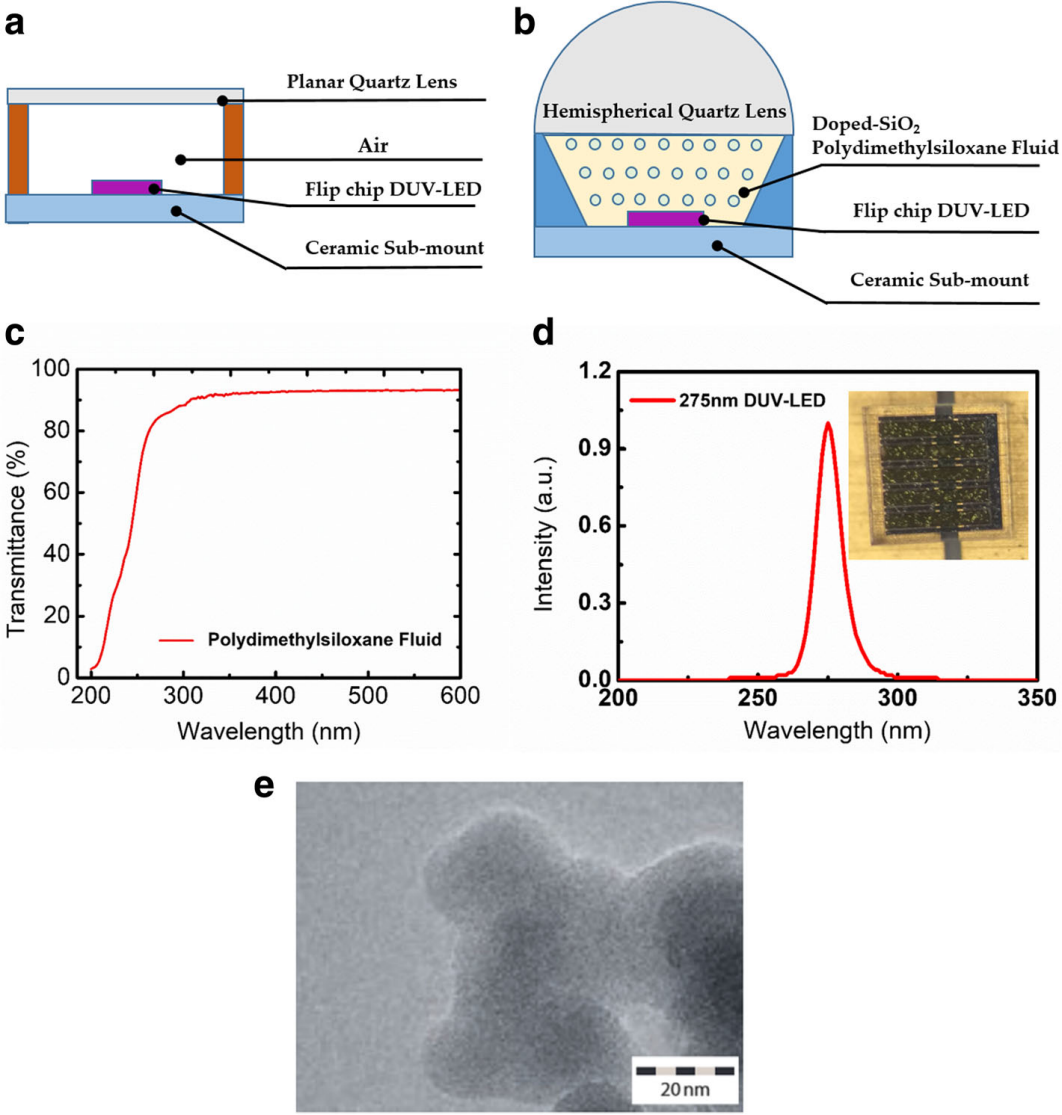
DUV-LED encapsulation structure: **a** Schematic of conventional flip chip DUV-LED, **b** encapsulation structure and SiO_2_ nanoparticle (NP)–doped polydimethylsiloxane (PDMS) fluid DUV-LED encapsulation structure, **c** transmittance of the PDMS fluid from 200–600 nm, **d** photograph of the DUV-LED and emission spectrum captured at a forward current of 200 mA for the proposed DUV-LED, and **e** high-resolution transmission electron microscopy image of SiO_2_ NPs^26^

Figure 1 shows the schematic of the proposed DUV-LED encapsulation process consisting of the following steps: (a) a ceramic substrate is prepared with alumina as the electrode material; (b) the DUV-LED chip (peak wavelength 275 nm) is bonded to the ceramic substrate through hot pressure bonding; (c) the aluminum reflector sidewall cavity is bonded to the DUV-LED ceramic substrate, and the chip is placed at the center of the opening; (d) PDMS fluid is dispensed into the aluminum reflector sidewall cavity; (e) coating binder and a hemispherical UV-transmissive glass with a diameter of 3 mm and height of 1.3 mm are placed on the outer ring of the aluminum reflector sidewall cavity; (f) individual DUV-LEDs are cut out along the scribe lines; and (g) a complete DUV-LED with a SiO_2_-NP-doped PDMS fluid encapsulation structure is obtained. Figure 2a illustrates a conventional DUV-LED, and Fig. 2b shows a DUV-LED encapsulated with PDMS fluid proposed in this study. The intermediate layer comprises PDMS doped with SiO_2_ NPs. The traditional method uses a vertical ceramic sidewall on the left- and right-hand sides of the DUV-LED flip chip, planar UV-transmissive glass on the top, and air as the medium between the DUV-LED flip chip and glass. The middle layer of the proposed design was an encapsulated structure of SiO_2_ NPs in PDMS fluid with a hemispherical UV-transmissive glass structure above. Figure 2c plots the transmittance of the PDMS fluid at different wavelengths as obtained using an optical spectrophotometer measurement system (Hitachi, Tokyo, Japan). The graph reveals that the PDMS fluid transmittance was 85% at 275 nm. Figure 2d presents a photograph of the DUV-LED with a surface area of 0.78 × 0.75 mm^2^ (Dowa Co. Ltd., Tokyo, Japan) and its emission spectrum was captured at 200 mA forward current. The chip’s dominant wavelength was 275 nm with a full width at half maximum of 12 nm. All data were obtained using an optical system SLM-20 integrating sphere (Isuzu Optics, Hsinchu, Taiwan). Table 1 lists the specifications (surface and material properties) of all the components of the proposed encapsulated DUV-LED.

**Table 1:** Materials and characteristics of DUV-LED

Component	Characteristics	Material
DUV-LED	Peak wavelength = 275 nm	AlGaN
Nanoparticles	Average primary particle size = 14 nm Refractive index (*n*) = 1.45 Energy gap (Eg) = 9 eV	SiO_2_
Tilt angle reflector sidewall cavity	Tilt angle = 60° Diameter = 2 mm	Aluminum
Hemispherical Quartz glass	Diameter = 4 mm High = 1.3 mm	Quartz
Encapsulation structure	Polished	Al_2_O_3_ ceramics
Encapsulation material	Refractive index (*n*) = 1.45	Polydimethylsiloxane fluid

A transmission electron microscopy image of the SiO_2_ NPs (AEROSIL hydrophobic fumed silica, Frankfurt am Main, Germany) is presented in Fig. 2e. The NPs were prepared by first removing the moisture at 150 °C in an oven and then placing the NPs in a N_2_ tank for 48 h to dry their surfaces. The average size of the NPs was set at 14 nm to prevent them from sticking together due to moisture.

## Results and Discussion

**Fig. 3:**
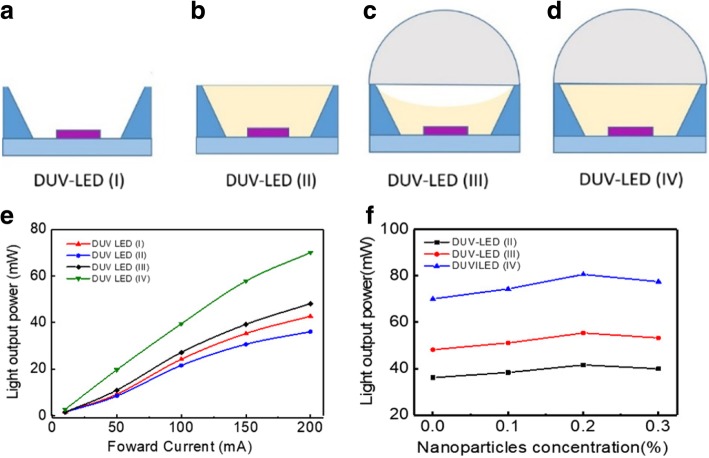
Schematic and comparison of the four encapsulation structures: **a** DUV-LED (I), **b** DUV-LED (II), **c** DUV-LED (III), **d** DUV-LED (IV), **e** light output power under different encapsulation conditions, and **f** light output power with different SiO_2_ NP concentrations (%) in the PDMS fluid

**Fig. 4:**
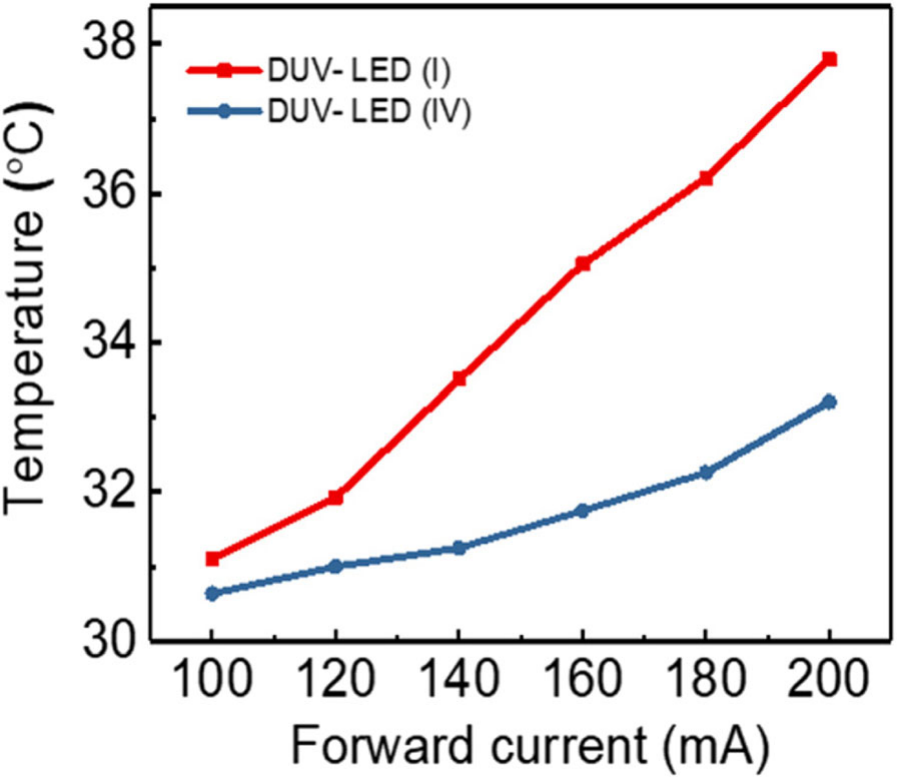
Average surface temperature dependence of DUV-LED (I) and DUV-LED (IV)

Four types of DUV-LED encapsulation were employed and are shown in Fig. 3. Figure 3a shows DUV-LED (I) with a DUV-LED chip and aluminum reflector sidewalls at an angle of 60°. Figure 3b shows DUV-LED (II) in which the aluminum reflector sidewall cavity was filled with PDMS fluid. Figure 3c shows DUV-LED (III) in which the aluminum reflector sidewall cavity was filled with slightly less PDMS fluid than that in DUV-LED (II) and with a hemispherical UV-transmissive glass cover. Figure 3d shows DUV-LED (IV) in which the aluminum reflector sidewall cavity was completely filled with PDMS fluid and a hemispherical UV-transmissive glass cover was used. Integrating sphere measurement was performed for the four types of DUV-LED encapsulation (Fig. 3e). When the driving current of the DUV-LED (I) chip was 200 mA, the light output power was 42.07 mW. By contrast, when the drive current of the DUV-LED (II) chip was 200 mA, the light output power was 36.11 mW, which was 14.16% lower than that for DUV-LED (I). This condition occurred mainly because TIR transpired when PDMS fluid filled the aluminum reflector sidewall cavity. The extraction efficiency ratio of UV light coupled into the PDMS fluid to UV light coupled into air is given by the following equation [12]:

1 $$ \frac{\eta_{PDMSfluid}}{\eta_{air}}=\frac{1-{\mathit{\cos}}_{\theta c, PDMS\kern0.5em fluid}}{1-{\mathit{\cos}}_{\theta c, air}}, $$

where *θ*_c,PDMS fluid_ and *θ*_c,air_ are the critical angles for TIR at the PDMS fluid DUV-LED and air UV-LED interfaces, respectively. When the driving current of the DUV-LED (III) chip was 200 mA, the optical output power was 48.126 mW, which was 14.39% higher than that for DUV-LED (I). This condition occurred mainly because the concave lens reduced the TIR but increased the LEE. However, DUV-LED (III) had an air gap, which hindered it from having the highest light output power among all the fabricated devices. When the driving current of the DUV-LED (IV) chip was 200 mA, the output power was 70.045 mW, which was 66.49% higher than that of DUV-LED (I). The DUV-LED (IV) encapsulation structure yielded the highest light output power because no air gap was present in the encapsulation, thus enabling the full transmission of DUV light from the DUV-LED. The light output power was also determined for DUV-LED (II), DUV-LED (III), and DUV (IV) encapsulation when the PDMS fluid was doped with SiO_2_ NPs (Fig. 3f). The DUV-LED (I) structure was not included in the comparison because it did not contain PDMS fluid. The weight percentage concentrations (%) of NP were set to 0, 0.1, 0.2, and 0.3 wt%. When the driving current of the DUV-LED (IV) chip was 200 mA, the light output power was 70.04, 74.32, 80.58, and 77.44 mW. Thus, a SiO_2_ NP doping concentration of 0.2 wt% resulted in the highest LEE. Doping the PDMS fluid with SiO_2_ NPs increased the amount of scattered light but decreased the amount of TIR. Doping with 0.2 wt% SiO_2_ NP resulted in 15% higher LEE than doping with 0 wt% SiO_2_ NP. Compared with that of DUV-LED (I), the LEE was 81.45% higher for a driving current of 200 mA. DUV-LED encapsulation was performed using the manufacturing methods outlined in Fig. 3. Table 2 shows the images of the operation at a driving current of 200 mA of the DUV-LED (IV) with PDMS fluid doping at 0.2 wt% SiO2 NPs. Figure 4 provides a comparison of the average interface temperatures of DUV-LED (I) and DUV-LED (IV) containing SiO_2_ NP-doped PDMS fluid at different driving currents. When the driving current was 200 mA, the interface temperature in the DUV-LED (IV) device was 4 °C lower than that in the DUV-LED (I) device, revealing that the encapsulation structure effectively weakened the thermal temperature. Table 2 shows a temperature map of the DUV-LED (I) and DUV-LED (IV) that was obtained using an infrared thermal imager (ChingHsing Co. Ltd., Taipei, Taiwan). At the driving current of 140 mA, the DUV-LED (IV) had lower operating temperature than the DUV-LED (I). For DUV-LED (I) without PDMS fluid, the temperature was the highest on the surface of the chip. The results in Fig. 4 and Table 2 reveal that the encapsulation structure with PDMS fluid doped with SiO_2_ NPs has superior heat dissipation capability.

**Table 2:**
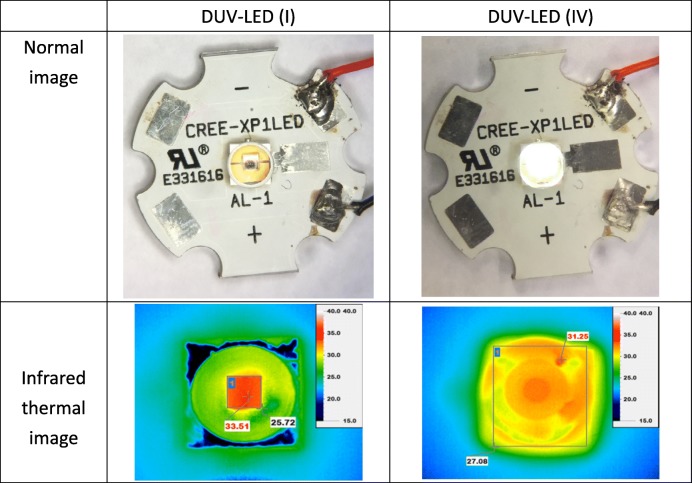
Image of DUV-LED (IV) containing PDMS fluid doped with SiO_2_ NPs and its operation. Infrared thermal images of (a) the normal image of DUV-LED (I), (b) the normal image of DUV-LED (IV) in normal image, (c) the infrared thermal image of DUV-LED (I), and (d) the infrared thermal image of DUV-LED (IV) in normal image

## Conclusions

This paper proposes a new encapsulation method for improving the LEE of DUV-LEDs by doping the PDMS fluid with SiO_2_ NPs. A considerably high light output power was achieved by using the SiO_2_ NP-doped PDMS fluid encapsulation structure. IN particular, the light output power was 81.45% higher when the PDMS fluid doped with 0.2 wt% SiO_2_ NPs was placed in the cavity rather than in the air. This enhancement is attributed to the reduced TIR and the additional light scattering in the PDMS fluid because of the addition of SiO_2_ NPs. The average interface temperature was 4 °C lower at a driving current of 200 mA. The proposed architecture was compact and feasible for fabricating high-LEE AlGaN-based DUV-LEDs in the future.

## Data Availability

Not applicable

## Abbreviations

DUV-LEDs: Deep-ultraviolet light-emitting diodes
NPs: Nanoparticles
PDMS: Polydimethylsiloxane
